# Immune Modulation by Microbiota and Its Possible Impact on Polyomavirus Infection

**DOI:** 10.3390/pathogens14080747

**Published:** 2025-07-30

**Authors:** Giorgia Cianci, Gloria Maini, Matteo Ferraresi, Giulia Pezzi, Daria Bortolotti, Sabrina Rizzo, Silvia Beltrami, Giovanna Schiuma

**Affiliations:** Department of Environmental and Prevention Sciences, University of Ferrara, 44121 Ferrara, Italy; giorgia.cianci@unife.it (G.C.); gloria.maini@unife.it (G.M.); matteo.ferraresi@unife.it (M.F.); giulia.pezzi@unife.it (G.P.); silvia.beltrami@unife.it (S.B.); giovanna.schiuma@unife.it (G.S.)

**Keywords:** polyomavirus pathogenesis, gut microbiota, immune modulation

## Abstract

Polyomaviruses are a family of small DNA viruses capable of establishing persistent infections, and they can pose significant pathogenic risks in immunocompromised hosts. While traditionally studied in the context of viral reactivation and immune suppression, recent evidence has highlighted the gut microbiota as a critical regulator of host immunity and viral pathogenesis. This review examines the complex interactions between polyomaviruses, the immune system, and intestinal microbiota, emphasizing the role of short-chain fatty acids (SCFAs) in modulating antiviral responses. We explore how dysbiosis may facilitate viral replication, reactivation, and immune escape and also consider how polyomavirus infection can, in turn, alter microbial composition. Particular attention is given to the Firmicutes/Bacteroidetes ratio as a potential biomarker of infection risk and immune status. Therapeutic strategies targeting the microbiota, including prebiotics, probiotics, and fecal microbiota transplantation (FMT), are discussed as innovative adjuncts to immune-based therapies. Understanding these tri-directional interactions may offer new avenues for mitigating disease severity and improving patient outcomes during viral reactivation.

## 1. Polyomaviruses

Polyomaviruses are a family of non-enveloped, double-stranded DNA (dsDNA) viruses [1]. The term “polyomavirus” originates from their capacity to induce multiple tumors in mouse models under specific conditions, a phenomenon first documented with the murine polyomavirus (MPyV) in the early 1950s [2]. Since then, species-specific polyomaviruses have been identified in a wide range of hosts, including primates, rodents, bats, cattle, sea lions, horses, raccoons, rabbits, and numerous bird species [3]. These viruses are also pathogenic to humans and have been detected at various body sites, such as in the blood, respiratory fluids, skin, liver, stool, gastrointestinal tract tissues, and in the central nervous system (CNS) [4].

The taxonomy of the *Polyomaviridae* family includes three genera: *Orthopolyomavirus* and *Wukipolyomavirus*, which contain mammalian species, and *Avipolyomavirus*, which includes avian species [5]. The *Orthopolyomavirus* genus also includes four human polyomaviruses that may be implicated in cancer developmen: Simian virus 40 (SV40), BK polyomavirus (BKPyV), JC polyomavirus (JCPyV), and Merkell cell polyomavirus (MCPyV) [6]. In addition, recent studies identified 11 additional polyomaviruses in human tissues and specimens (Table 1).

While BKPyV, JCPyV, TSPyV, and MCPyV are associated with specific human diseases, the clinical implications of new human polyomaviruses remain only partially understood; therefore, further investigations are needed [19].

### 1.1. Genome, Structure, and Replication of Polyomaviruses

**Figure 1:**
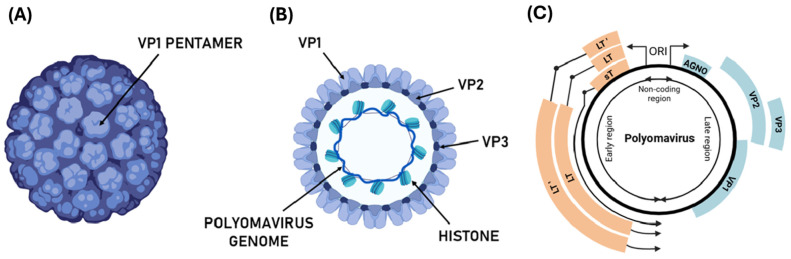
**Structure and genome organization of the polyomavirus**. (**A**) Schematic representation of the polyomavirus virion, showing the icosahedral capsid composed of VP1 pentamers; (**B**) representation of a section of polyomavirus virion and the circular dsDNA genome with histones and structural protein VP1, VP2, and VP3; (**C**) representation of polyomavirus genome organized into early (orange genes), late (blue genes), and non-coding control regions (NCCR). The genome organization in LT, LT’ (alternatively-spliced LT antigens), and sT, genes encoding regulatory and structural (Agnoprotein—AGNO—VP1, VP2, VP3) are indicated.

Human polyomaviruses are characterized by a circular, covalently closed, supercoiled dsDNA genome of about 5000 bp [20]. Viral DNA is packaged with histone proteins, forming a minichromosomal structure (Figure 1). It consists of three functional regions: early, late, and a noncoding control region (NCCR), which separates the early and late regions. The NCCR region includes the origin of replication, with several transcription regulatory sequences for both early and late regions [21].

The early transcription region contains genomic sequences expressed during early infection, such as the large T antigen (LT, ~80 kDa) and the small T antigen (sT, ~20 kDa). These antigens are produced through a process of alternative splicing, where the 5′ end of the RNA transcript serves as a common starting point for both antigens, while they differ at their 3′ ends [22]. The late transcription region is activated in the final stage of viral replication and typically supports the production of three structural capsid proteins: VP1, VP2, and VP3 [23]. In certain polyomaviruses, an additional protein, VP4, has also been identified. Proteins encoded in the early region tend to show greater similarity across different species than those encoded in the late region [24].

Interestingly, while the late region of the BKPyV, JCPyV, and SV40 genomes encode agnoprotein—a small regulatory phosphoprotein [25,26], the MCPyV genome lacks this gene.

Agnoprotein (62–71 amino acids) enhances viral stability and propagation via phosphorylation [26,27] and is essential for efficient DNA replication, transcription, and virion assembly. It interacts with viral proteins (e.g., LT, sT, VP1) and host factors (e.g., p53, Ku70, PP2A, YB-1), modulating key steps in the viral life cycle [25,26,27].

Functioning as a viroporin, agnoprotein increases membrane permeability, facilitating virion release and disrupting host calcium homeostasis, which may impair the cell cycle and DNA repair [28,29,30,31].

Although its role in gut microbiota interaction remains unclear, its effects on epithelial barrier integrity and membrane permeability suggest it may influence host–microbe dynamics during intestinal infections [32].

Agnoprotein-driven immune modulation may alter microbiota composition by affecting local immune responses and triggering inflammation through increased membrane permeability and calcium imbalance [32]. Its involvement in chemokine regulation further suggests a role in immune evasion and viral persistence [33], potentially linking agnoprotein to long-term immune disruption and microbial imbalance.

Thus, through its viroporin activity and regulatory functions, agnoprotein may contribute to both viral pathogenesis and host–microbiota–immune interactions.

Structurally, the assembly of polyomavirus capsid involves five VP1 proteins forming a capsomer (VP1 pentamer), which interacts with other units to form the viral capsid composed of 72 pentameric capsomers. This results in virions approximately 40–45 nm in diameter, with an icosahedral shape [34]. The structural integrity of the viral capsid is maintained by interaction between VP1 molecules, with the C-terminal residue of VP1 interacting with the adjacent capsid protein. The major capsid component, VP1, is associated with two minor capsid proteins, VP2 and VP3, which are expressed in mature viral particles and positioned at the center of the pentamer structure [35] (Figure 1).

The species-specific infectivity of polyomaviruses is largely attributed to the interaction between the VP1 protein and sialic acids on host cell membranes, serving as the target receptors [36].

Polyomavirus replication occurs in two principal steps [37]: (i) the early stage, from virus binding to the host cell receptor through viral DNA replication, and (ii) the late stage, which includes several events enabling assembly and release of new virions. Replication begins with the adsorption of virions to the host cell surface, triggering the cytoplasmic entry of viral particles. This process is facilitated by enzymatic modifications of the host cell receptor. BKPyV and JCPyV employ distinct mechanisms for internalization: BKPyV enters via caveolae-mediated endocytosis—a clathrin-independent endocytosis that involves membrane invaginations known as caveolae [38], while JCPyV enters cells via clathrin-dependent endocytosis [39].

Subsequently, the polyomavirus undergoes genome uncoating, and the viral DNA enters the nucleus of the cell. There, the virus activates the transcription of early genes (LT and sT antigens), followed by expression of late genes (VP1, VP2, and VP3), facilitating assembly of the viral capsid. The final phase is marked by the release of newly formed virions, leading to host cell lysis [30].

### 1.2. Pathogenesis and Clinical Relevance of Polyomaviruses

Polyomaviruses exhibit unique pathophysiological features and clinical relevance, particularly in immunocompromised individuals, where they can cause considerable morbidity and mortality. The high susceptibility to polyomavirus-associated pathologies underscores the crucial role of the host immune response during these infections [40].

BKPyV infection is often asymptomatic in healthy individuals, but it can cause severe complications in kidney transplant patients, leading to significant inflammation and tissue damage [41]. For example, a study by Gately et al. found that BKPyV is a leading cause of graft loss in kidney transplants, with 1–10% of transplant patients developing BKPyV-associated nephropathy (BKPyVAN) [42]. The virus primarily targets renal tubular epithelial cells, resulting in viral spread in urine and blood. Under immunosuppressive conditions, BKPyV reactivates, inducing nephropathy and hemorrhagic cystitis (HC), with symptoms including hematuria, dysuria, and elevated serum creatinine levels [43].

In contrast, JCPyV is associated with progressive multifocal leukoencephalopathy (PML), a severe demyelinating disease affecting the CNS [44]. After primary infection, the virus remains latent in the kidneys and lymphoid tissues, reactivating during immunosuppression. JCPyV specifically infects oligodendrocytes in the brain, leading to their destruction and resulting in neurological symptoms. PML poses a significant risk for patients undergoing immunosuppressive therapies, such as those with multiple sclerosis or organ transplants [45].

Among polyomaviruses related to human diseases, MCPyV is primarily associated with Merkel cell carcinoma (MCC), a rare and aggressive skin cancer [46]. MCPyV can integrate into the host genome, resulting in the high expression of oncogenic proteins that disrupt normal cellular functions and favor malignant transformation [47].

Recent studies also suggest that these polyomaviruses may be implicated in gastrointestinal tract cancers [48]. For instance, JCPyV and BKPyV have been detected in colorectal cancer tissues [19], but the precise role of these viruses in cancer progression remains unclear, warranting further investigation [40].

### 1.3. Host Immune Response to Polyomavirus Infection

The interaction between polyomaviruses and the immune system is multifaceted, involving both innate and adaptive responses, as well as immune evasion strategies. Despite the role of the innate immune system in recognizing viral infections, polyomaviruses have developed distinct mechanisms to escape these defenses [49,50]. For example, BKPyV disrupts mitochondrial pathways, impairing interferon regulatory factor 3 (IRF3) signaling and interferon-β (IFN-β) production, which are crucial for initiating antiviral states in infected cells [50]. BKPyV’s VP1 protein also impairs dendritic cell (DC) maturation, which is essential for presenting viral antigens to T cells and generating BKPyV-specific T lymphocytes [51]. This reduced adaptive immune response enhances viral replication [52]. Meanwhile, MCPyV downregulates Toll-like receptor 9 (TLR9) to prevent the recruitment of immune cells and the activation of inflammatory responses [53].

Studies in human astrocytes, the main target cells for the JCPyV, show that both retinoic acid-inducible gene I (RIG-I) and cyclic GMP-AMP synthase (cGAS) are essential in reducing JCPyV replication [54]. Type I interferons, such as IFN-β, are central in counteracting JCPyV infection, although the virus has evolved strategies to suppress these pathways [55,56].

Adaptive immunity is also critical in controlling polyomavirus infections, as neutralizing antibodies target the VP1 capsid protein [57]. Several studies show that BKPyV-specific CD8^+^ and CD4^+^ T cells, as well as IgG antibodies, increase in post-transplant positive patients [58]. In mouse models, JCPyV evades neutralizing antibodies by mutating its VP1 protein, enabling more effective CNS infection [59]. The control of JCPyV infection may also be influenced by specific human leukocyte antigen (HLA) class II variants, which are associated with varying susceptibility [60].

Polyomavirus reactivation in immunosuppressive conditions—such as during Tacrolimus and Mycophenolate mofetil treatments—occurs due to immune modulation, particularly through impairment of T lymphocyte activation. This suppression diminishes cytokine production (e.g., interferon-gamma, interferon-γ (IFN-γ) and interleukin-2 (IL-2)), weakening antiviral defenses [45,61]. While polyomaviruses employ sophisticated immune evasion strategies, their interaction with host immunity may involve crosstalk with the gut microbiota, which is fundamental for maintaining systemic immunity and antiviral pathways.

## 2. Role of the Gut Microbiota in Immune Homeostasis and Possible Effects on Polyomavirus Infections

### 2.1. Gut Microbiota Characterization

**Figure 2:**
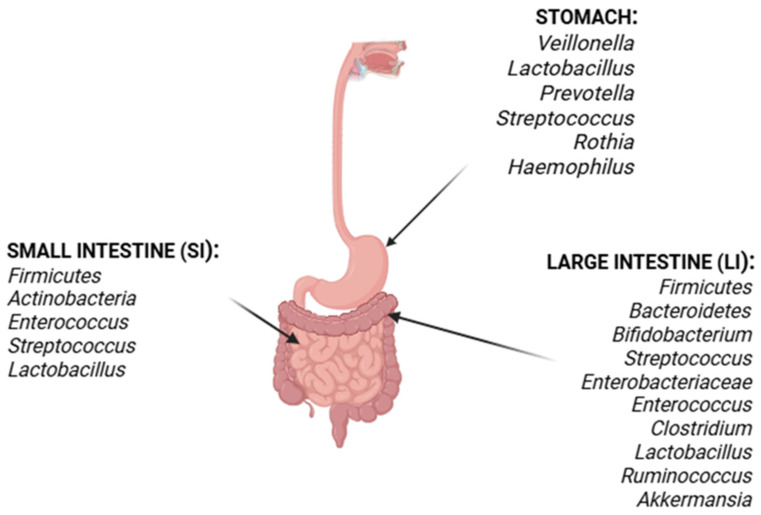
**Distribution of microbiota along the human gastrointestinal tract.** Illustration of gut microbiota composition across anatomical sections from stomach to colon, highlighting predominant phyla such as Firmicutes and Bacteroidetes and their relative abundance along the intestinal segments.

The human gut, covering 200–300 m^2^ of mucosa, harbors around ten trillion diverse symbionts, collectively known as the “microbiota”. This complex system includes bacteria, archaea, eukaryotes, viruses, and parasites [62,63,64]. These numerous species are distributed differently across body sites. The digestive tract is colonized by the phyla *Firmicutes*, *Bacteroidetes*, *Actinobacteria*, *Fusobacteria*, *Cyanobacteria*, *Verrucomicrobia*, and *Proteobacteria* [65] (Figure 2), with *Bacteroidetes* and *Firmicutes* constituting more than 90% of the total population.

The gastrointestinal tract is divided anatomically and functionally into the stomach, small intestine (SI), and large intestine (LI) (Figure 2). Each section displays its own physiological barrier and microenvironment, allowing specific microorganisms to colonize these regions [66]. The microbial community in the human stomach consists of acid-resistant oral species, like *Veillonella*, *Lactobacillus*, *Prevotella*, *Streptococcus*, *Rothia*, and *Haemophilus* [67].

Following the stomach, the SI is divided into three parts: the duodenum, jejunum, and ileum. The duodenum’s microenvironment is characterized by bile acids, pancreatic secretions, and antimicrobial agents, with *Firmicutes* and *Actinobacteria* predominating [68]. The jejunum is distinguished by Gram-positive aerobes and facultative anaerobes, including *Lactobacillus*, *Enterococcus*, and *Streptococcus* [69]. As bacterial density increases in the ileum, aerobic species, anaerobes and Gram-negative organisms become more prevalent, with the highest density in the colon [70] (Figure 2).

The LI comprises the ascending, transverse, and descending colon, as well as the cecum. This section is predominantly anaerobic, with *Firmicutes* and *Bacteroidetes* as the major phyla represented. The most prevalent genera in this region include *Bifidobacterium*, *Streptococcus*, *Enterobacteriaceae*, *Enterococcus*, *Clostridium*, *Lactobacillus*, and *Ruminococcus*; notably, *Clostridium, Lactobacillus, Enterococcus*, and *Akkermansia* are also present in significant numbers [71,72].

The composition of the gut microbiota is influenced by multiple factors, including genetics, diet, age, and environmental exposures [73]. These factors contribute to the dynamic and individualized nature of the microbial community, which plays a crucial role in maintaining host health.

As can be observed, the two dominant bacterial phyla in the gut microbiota are *Firmicutes* and *Bacteroidetes*. The ratio between these two groups is considered a predictive marker for health and disease. Several studies indicate that an imbalance in the *Firmicutes*/*Bacteroidetes* ratio is associated with obesity, inflammatory bowel disease, and metabolic disorders [74].

Notably, the microbiome encompasses not only the microorganisms themselves, but also their genomes, metabolites, and proteomes, which together reflect the biological environment [75]. A wide range of metabolites are produced through both anaerobic fermentation of exogenous dietary components (food) and endogenous compounds generated by microorganisms and the host, such as short-chain fatty acids (SCFAs) [76].

### 2.2. The Gut Virome

As mentioned above, the human gut microbiota is significantly variegated. In fact, in addition to the bacterial population, several viruses have been identified as part of the human microbiota. The term “gut virome” refers to the collection of viruses that colonize the intestine, with viral particles often outnumbering bacterial cells (1:1 to 10:1 ratio) [77,78]. Similar to the bacterial microbiome, alterations in the gut virome can impact the development of several diseases, including inflammatory bowel disease (IBD) [79], obesity, diabetes [80], liver diseases [81], colorectal cancer (CRC), and malnutrition [82,83].

During IBD, changes in the virome are related to the expansion of *Caudovirales bacteriophages*, such as *Siphoviridae* or *Podoviridae*, and a reduction in viral diversity [84,85]. Dysregulated phages can exacerbate colitis by disrupting epithelial barrier integrity, promoting proinflammatory responses, and altering bacterial populations like *Bacteroidetes* [84].

In type 2 diabetes and diabetic nephropathy, there is a decrease in viral richness and diversity, depletion of *Flavobacterium* phages and *Bacteroides* phages, along with enrichment of *Shigella* phages. These virome changes reduce phage lysing activity and disrupt bacterial regulation, contributing to metabolic dysfunction [85,86].

In CRC, elevated *Caudovirales* phages and disrupted viral metabolic pathways (e.g., reduced L-methionine and acetate production) promote oncogenesis by weakening tumor-suppressive metabolites and altering host–microbe interactions [84,87].

The role of polyomaviruses in the gut virome remains poorly characterized compared to other viral components, like bacteriophages. Available evidence from studies of human fecal samples suggests that polyomaviruses (e.g., MW polyomavirus and MX polyomavirus) have been identified in stool samples, but their prevalence and abundance are low compared to those of dominant gut viruses like *Caudoviricetes* bacteriophages or pepper mild mottle virus [88].

No direct causal relationship has been established between polyomavirus presence and specific gut physiological processes, immune modulation, or disease states. In contrast, the gut virome is primarily shaped by bacteriophages, which regulate bacterial populations, through predation and gene transfer, and eukaryotic viruses linked to dietary or environmental exposure [89].

While polyomaviruses are part of the broader virome, their interactions with host cells, bacteria, or immune pathways in the gut remain unstudied. Current research focuses on their detection rather than on their mechanistic roles, highlighting a gap in understanding their potential contributions to homeostasis or dysbiosis in the intestinal tract [90,91].

Furthermore, the human intestinal tract contains the largest concentration and diversity of immune cells among all body organs, through which the brain and gastrointestinal tract are in close contact [92]. Thus, the intestinal microbiota is strongly implicated in the modulation of the immune system, aimed at protecting against pathogens and related infections. The interaction between gut microbiota and host immunity is complex and dynamic, involving both innate and adaptive immune components [93].

### 2.3. Role of Gut Microbiota in Host Physiopathology

The complex interplay between resident gut microbiota and the host is at the base of crucial changes at both the tissue and immune system level. The gut microbiota plays a fundamental role in maintaining host physiology and health by contributing to nutrient metabolism, immune system development, and protection against pathogens [94].

It performs essential functions such as fermenting indigestible dietary fibers, synthesizing vitamins, and producing bioactive metabolites that influence systemic processes. Importantly, it affects insulin secretion and consequently modulates both insulin resistance and sensitivity [95]. Notably, alterations in the gut microbiota are associated with both type 1 and type 2 diabetes, impairing metabolite production and immune–inflammatory pathways [96].

One of the key contributions of the gut microbiota is the production of microbial metabolites such as short-chain fatty acids (SCFAs)—including acetate, propionate, and butyrate—through the fermentation of dietary fibers [97,98]. SCFAs serve as energy sources for colonocytes, help maintain intestinal barrier integrity, and modulate immune responses by promoting anti-inflammatory pathways and the differentiation of regulatory T cells. These effects support mucosal homeostasis and prevent excessive inflammation [99]. For instance, reduced SCFAs levels compromise intestinal barrier function, allowing the release of bacterial products (such as lipopolysaccharides (LPSs)) and triggering inflammation. This correlation has been observed in patients with type 1 diabetes, who exhibit reduced SCFAs levels [100,101].

Furthermore, the gut microbiota is implicated in obesity, non-alcoholic fatty liver disease (NAFLD), insulin resistance, and chronic inflammation, all of which are strongly correlated with the development of type 2 diabetes [102]. Obese individuals typically exhibit a reduced abundance of gut microbiota, and NAFLD patients show lower *Oscillospira* abundance and elevated concentrations of 2-butanone and 1-pentanol.

The composition of gut microbiota can also be modulated by dietary factors, such as high-fat and high-fructose intake. Supporting this notion, dietary interventions—such as the Mediterranean diet, which is rich in fiber and omega-3 fatty acids—are used to restore microbial balance, enhancing SCFAs production and reducing inflammation [100].

Moreover, bacteria within the gut microbiota regulate the proliferation and differentiation of intestinal epithelial cells, influencing brain–gut communication and neurological function in the host [103]. The gut–brain axis (GBA) refers to the bidirectional communication network between the brain and the intestine, regulating endocrine pathways, gut motility, and intestinal permeability [104,105]. The association between intestinal–hormonal pathologies—such as obesity—and the GBA has been well established, as gut microbiota modulates the secretion of hormones such as glucagon-like peptide-1 (GLP-1), ghrelin, peptide YY, and leptin [106]. Intestinal bacteria also synthesize neuroactive metabolites (e.g., serotonin and gamma-aminobutyric acid (GABA)), which influence appetite regulation by the central nervous system. Serotonin contributes to reduced food intake, whereas GABA plays a crucial role in maintaining energy balance [107], highlighting their importance in brain–gut communication. Recently, the role of gut microbiota has been described not only in regards to the influence of the brain–gut axis in association with dietary and hormone function, but its effect has also been reported to be crucial in the regulation of host immune response.

### 2.4. Gut Microbiota and Immune System Interactions

The gut microbiota also influences the development and function of the host immune system. It educates immune cells, promotes immune tolerance, and enhances the host’s ability to respond to infections.

Commensal bacteria stimulate the production of antimicrobial peptides and support the maturation of gut-associated lymphoid tissue (GALT), which is critical for mounting appropriate immune responses and maintaining immune homeostasis [108,109].

Innate immunity serves as the first line of defense against bacterial and viral infections, involving physical barriers [110,111] and pattern recognition receptors (PRRs) [112] to prevent pathogen invasion. Within this context, PRRs such as Toll-like receptors (TLRs) are essential for detecting pathogen-associated molecular patterns (PAMPs) derived from microbes. TLRs activate signaling pathways such as nuclear factor-κB (NF-κB). Paneth cells—specialized epithelial cells in the small intestine—further contribute by secreting defensins and cathelicidins in response to TLR activation. These mechanisms help shape the microbiota composition and enhance pathogen resistance [113]. Natural killer (NK) cells also play a pivotal role in innate immunity within the gut, interacting with epithelial and immune cells to maintain homeostasis and produce antiviral IFN-γ. Dysregulation of NK cell activity can lead to inflammatory conditions such as IBD.

Phagocytes, including macrophages and DCs, are integral to innate immunity in the gut. These cells eliminate pathogens via phagocytosis and express PRRs to detect microbial threats, thereby triggering inflammatory responses and facilitating pathogen clearance. Together, PRRs, NK cells, and phagocytes form a complex system that maintains intestinal homeostasis [114].

The gut microbiota participates not only in the modulation of innate immunity, but also in the regulation of the adaptive immune system [115]. Notably, bacteria from the intestinal microbiome significantly influence the adaptive immune response by affecting T cell maturation, differentiation, and activity. The interplay between T helper cells (Th1, Th2, and Th17) and regulatory T cells (Tregs) is finely regulated by microbially derived metabolites [116]. Microbial metabolites, such as SCFAs from commensal bacteria, directly influence DCs functions by modulating their cytokine production and surface receptor expression. For instance, Th1 cells are promoted by DC-derived interleukin-12 (IL-12), which is enhanced by microbial ligands such as LPS [117]. Tregs, which are critical for suppressing excessive inflammation, are induced by DCs in a TGF-β-rich environment and further potentiated by microbial metabolites like butyrate and propionate [118].

Moreover, the gut microbiota plays a critical role in B cell immunity through its interaction with GALT. GALT, located along the gastrointestinal tract, presents germinal centers as sites for B cell maturation and antibody production [119]. These B cells not only support gut immunity, but also circulate to other organs for systemic defense. In addition, the GALT–B cell relationship promotes the formation of immunoglobulin A (IgA)-secreting plasma cells, which are crucial for mucosal immunity [120]. Gut commensals also modulate B cell functions by inducing regulatory B cells (Bregs) that secrete interleukin-10 (IL-10), an anti-inflammatory cytokine, and by shaping IgA responses through both T cell-dependent and T cell-independent pathways [121]. Thus, the ability of gut microbes to influence immune cell response supports the role of dysbiosis in affecting host immune response.

### 2.5. Dysbiosis and Immune Modulation

Dysbiosis refers to an imbalance in the gut microbiota, characterized by alterations in the abundance or diversity of microbial species. This imbalance can disrupt immune homeostasis and has been linked to a variety of diseases, including inflammatory bowel disease, metabolic disorders, and increased susceptibility to infections.

Dysbiosis of gut microbiota can be driven by a variety of extrinsic and intrinsic factors, including diet, antibiotic exposure, allergen contact, hygiene practices, psychosocial stress, and host-specific variables. Dietary influence on microbiota is particularly profound; high-sugar, low-fiber diets disrupt the gut epithelial barrier, promote inflammation, and selectively shape the microbial community [122]. Moreover, antibiotic administration substantially depletes microbial diversity, increasing susceptibility to opportunistic pathogens [123,124]. Host factors—such as genetic predisposition, immune competence, comorbidities, and lifestyle—further modulate the gut microbial landscape [125,126,127]. Thus, a complex interplay of environmental and host-derived factors governs gut microbiota composition. Clinical observations have documented shifts in bacterial populations post-transplantation, typified by reductions in beneficial taxa (e.g., *Faecalibacterium prausnitzii*) and expansions of pathogenic species (e.g., *Escherichia* and *Shigella* spp.), contributing to a heightened risk of infection and gastrointestinal dysfunction [128,129].

In the context of polyomavirus infection, dysbiosis may facilitate viral replication and reactivation by impairing the host’s immune defenses. Several studies have demonstrated that patients with BK polyomavirus infection exhibit significant changes in their gut microbiota, particularly an increased Firmicutes/Bacteroidetes ratio. This shift is associated with immune deficiency and may serve as a potential biomarker for infection risk and immune status. Specific bacterial taxa, such as Romboutsia and Actinomyces, have been identified as distinguishing features in BKPyV-infected individuals, suggesting their potential as diagnostic markers.

Short-chain fatty acids (SCFAs), produced by gut bacteria through the fermentation of dietary fibers, play a pivotal role in modulating immune responses. SCFAs enhance the function of regulatory T cells and promote anti-inflammatory pathways, thereby contributing to the control of viral infections. Conversely, a reduction in SCFA-producing bacteria may weaken these protective immune mechanisms, increasing the risk of viral reactivation and disease progression.

Disruptions in gut microbiota composition can impair these physiological functions, leading to increased susceptibility to infections, chronic inflammation, and metabolic disorders. Therefore, maintaining a balanced and diverse gut microbiota is essential for preserving host health and resilience against diseases, including viral infections such as those caused by polyomaviruses [130,131].

## 3. How Polyomavirus Infection Could Impact on Intestinal Microbiota

### 3.1. Potential Role of Polyomaviruses in Microbiota Dysbiosis

**Figure 3:**
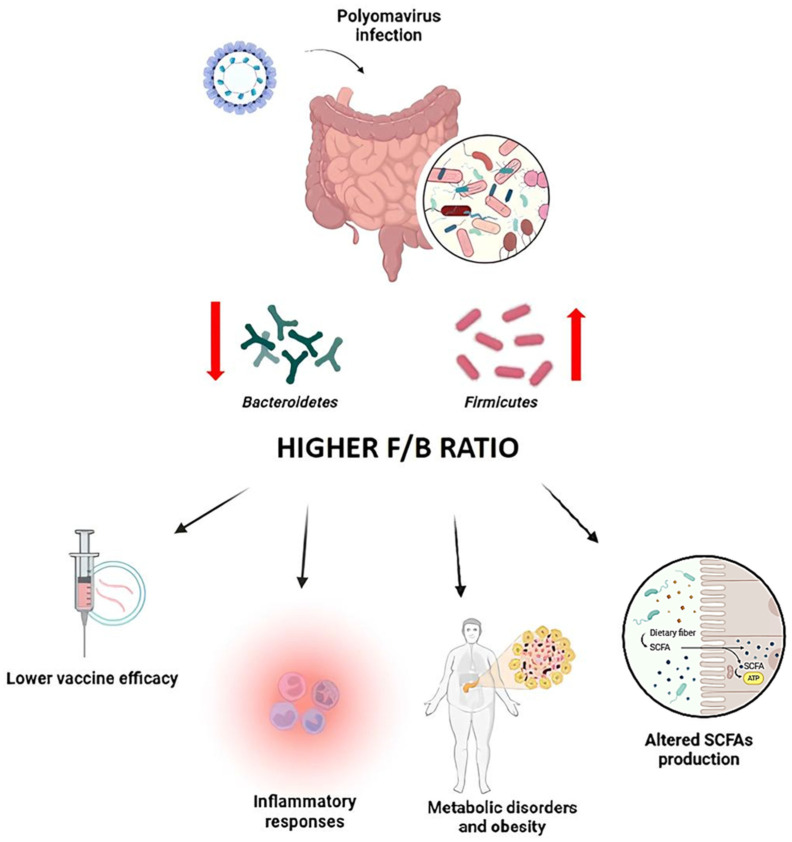
Impact of polyomavirus infection on the *Firmicutes*/*Bacteroidetes* (F/B) ratio and immune outcomes. Diagram showing how polyomavirus infection alters gut microbiota composition, increasing the F/B ratio, reducing microbial diversity, and impairing SCFA-mediated immune modulation, ultimately enhancing viral susceptibility and inflammation.

Given the importance of gut microbiota in modulating both innate and adaptive immune responses, its role in maintaining immune homeostasis during polyomavirus infections has gained increasing attention. The gut microbiota indirectly influences polyomavirus control by producing metabolites [132,133]. Polyomaviruses can directly interact with surface polysaccharides of specific gut-resident bacteria, such as bacterial LPS [134], thereby facilitating viral entry into host cells via the polyomavirus receptor (PVR) [135]. This interaction not only enhances viral infection and replication but also modulates the surrounding microbial ecosystem (Figure 3).

Virus-induced dysbiosis, in turn, compromises mucosal immunity and predisposes hosts to secondary infections [90]. Furthermore, alterations in microbial metabolite production, consequent to microbiota disruption, may exacerbate immune dysfunctions, amplifying the severity of viral pathogenesis. Several studies have documented a marked decline in microbial diversity among polyomavirus-infected patients, characterized by the overrepresentation of opportunistic pathogens and a corresponding deterioration in host health status [90,135].

Among the taxa most affected by viral infection are members of the phylum *Bacteroidetes*, which play a pivotal role in maintaining immune homeostasis. These bacteria are integral to T cell regulation and support the differentiation of Tregs, thereby curbing excessive inflammatory responses during infections, including those driven by polyomavirus [136,137]. A reduction in *Bacteroidetes* abundance often leads to an elevated Firmicutes/Bacteroidetes (F/B) ratio, impairing these protective mechanisms and fueling a proinflammatory milieu (Figure 3).

Polyomavirus infections have been shown to disrupt the *Firmicutes*/*Bacteroidetes* balance, promoting a dysbiotic state characterized by an increased F/B ratio [134,138]. Polyomavirus interactions with select bacterial species may preferentially support the proliferation of *Firmicutes* at the expense of *Bacteroidetes*, a shift commonly linked to heightened inflammatory responses. This microbial imbalance may exacerbate viral pathogenesis, diminish vaccine responsiveness [134,138], and contribute to broader dysbiotic consequences [134,139].

Importantly, the proinflammatory cytokine milieu produced in response to dysbiosis may further compromise immune defenses, amplifying tissue damage and worsening clinical outcomes (Figure 3) [134,140].

Beyond immune modulation, shifts in the F/B ratio also have significant metabolic implications. Elevated F/B ratios are associated with obesity and metabolic dysfunctions, phenomena that have been observed during polyomavirus infections (Figure 3) [141]. An excess of *Firmicutes* can alter SCFAs production, reduce anti-inflammatory effects, and negatively impact intestinal barrier function (Figure 3) [142]. These changes may further compromise the host’s ability to control viral replication and maintain immune balance, highlighting the intricate interplay between viral infection, microbiota composition, and host health [140].

The F/B ratio further influences vaccine efficacy. Higher *Bacteroidetes* abundance has been associated with enhanced antibody responses to vaccination, including those targeting polyomavirus antigens [137,143]. In contrast, individuals with a microbiota dominated by *Firmicutes* tend to exhibit lower vaccine-induced antibody titers, suggesting that dysbiosis may impair immunogenicity [137,138]. These findings highlight the importance of maintaining a balanced microbiota to optimize vaccine responses (Figure 3).

Given these insights, therapeutic modulation of the gut microbiota—through strategies to restore *Bacteroidetes* populations and rebalance the F/B ratio—may represent a promising adjunctive approach to enhance immune responses and mitigate the severity of polyomavirus infection [144].

In addition to the intestinal microbiota, the human body also harbors distinct microbial communities at other mucosal sites, including the ocular surface. Recent discoveries have demonstrated the presence of a specific ocular microbiome.

Of particular interest is the discovery of elevated MCPyV levels in the anophthalmic conjunctiva, shedding light on polyomavirus persistence in previously underexplored mucosal environments. In a study conducted by Siegal et al., researchers examined 20 anophthalmic sockets and their intact fellow eyes, detecting MCPyV in 19 out of 20 sockets, compared to only 5 out of 20 control sockets. Quantitatively, the viral load averaged 891 copies/ng in anophthalmic conjunctiva versus 193 copies/ng in intact eyes [145]. These results suggest that anatomical alterations may disrupt local homeostasis, facilitating viral expansion.

While the bacterial composition of the ocular microbiome remained relatively stable across conditions, the viral component—including MCPyV—underwent significant changes. Given that MCPyV is also detected in the gastrointestinal tract, lymphoid tissues, urine, and saliva, it appears to be a stable component of the resident human virome [145]. The systemic distribution of MCPyV raises the possibility that gut microbial ecology may influence viral dynamics at distant mucosal sites, such as the conjunctiva, via immune-mediated pathways.

### 3.2. Dysbiosis of Microbiota and BKPYV Susceptibility in Immunocompromised Patients

Emerging evidence suggests that dysbiosis may influence susceptibility to viral infections, including polyomavirus reactivation [125,129,146,147]. Notably, experimental models have demonstrated that antibiotic-induced microbiota depletion impairs viral clearance, facilitating polyomavirus persistence [148]. Furthermore, the combination of immunosuppressive therapy, altered dietary patterns, and frequent antibiotic use exacerbates dysbiosis and magnifies infection risk [149].

This phenomenon is particularly relevant in transplant recipients and immunocompromised individuals. Immunosuppression, whether pharmacologically induced or disease-related, renders these populations highly vulnerable to BKPYV reactivations [149].

Susceptibility to BKPyV infection and its complications, such as BKPyVAN, is shaped by both innate and adaptive immune responses [150].

Although BKPyV typically remains latent in immunocompetent hosts [151], reactivation is common under conditions of immunosuppression, particularly following organ transplantation [40]. Multiple factors, including recipient age, prior graft rejection episodes, and pre-existing BKPyV serostatus, impact clinical risk [152]. Genetic predispositions—such as variation in HLA-C and NCCR—as well as the presence of permissive tissues and local tissue injury, common in renal allografts, can facilitate viral replication and viremia, determining susceptibility and disease severity [52,153,154].

The type and intensity of immunosuppressive regimens significantly influence BKPyV susceptibility [150]. Excessive immunosuppression, particularly via agents such as Tacrolimus and Mycophenolate mofetil, correlates with higher incidences of BKPyV reactivation [152,155]. Concomitantly, these therapies exert profound effects on gut microbiota composition, disrupting microbial metabolic functions and diversity. Immunosuppressive agents can impair SCFAs production by gut bacteria, critical for immune modulation and gut integrity [128]. Reductions in SCFAs levels may, in turn, dysregulate immune homeostasis, enhance inflammatory responses, and increase graft rejection risk. For instance, Tacrolimus has been associated with diminished microbial production of SCFAs such as butyrate and propionate [156,157].

In transplant populations, microbial dysbiosis is not only linked to heightened viral susceptibility but also to broader clinical complications, including graft-versus-host disease (GVHD) and acute rejection episodes [158]. Importantly, dysbiosis correlates with poorer graft survival outcomes, emphasizing the potential significance of microbiota preservation strategies to improve long-term transplantation success [156,157,159].

However, the mechanistic underpinnings linking gut microbial alterations to viral reactivation remain incompletely understood, warranting further investigation into microbiome-targeted therapeutic approaches.

## 4. Polyomavirus Reactivation and Possible Effects on Microbiota-Mediated Immunity

As previously mentioned, the reactivation of polyomavirus observed in immunocompromised hosts (e.g., transplant patients) results from impaired T cell-mediated immune surveillance, allowing replication of latent viruses, such as BKPyV and JCPyV [160]. In addition, these viruses employ strategies to evade host defenses, such as using microRNAs (miRNAs) to suppress interferon signaling, which is fundamental to the antiviral response [161].

Moreover, commensal microbiota are known to stimulate immune pathways that are critical for counteracting polyomavirus reactivation. For example, SCFAs produced by the gut microbiota play a pivotal role in supporting Treg function and promoting an anti-inflammatory environment [162]. A decrease in SCFA-producing bacteria, often associated with dysbiosis, can impair immune responses and potentially contribute to the reactivation of latent viruses such as BKPyV. Moreover, the presence or absence of specific bacterial genera, including *Romboutsia* and *Roseburia*, in the gut microbiome of BKPyV-infected individuals may reflect the microbiota’s role in viral reactivation [163]. In patients with BKPyV infection, gut microbiota dysbiosis has been characterized by an increased *Firmicutes*/*Bacteroidetes* ratio. Similarly, *Bacteroidetes* abundance has been positively correlated with the CD4/CD8 ratio in individuals with HIV infection [90]. The CD4/CD8 ratio, calculated by dividing the number of CD4^+^ T cells by the number of CD8^+^ T cells, typically ranges between 1 and 3 in healthy individuals, with an average around 2:1 [164]. A higher CD4/CD8 ratio is generally associated with reduced chronic inflammation and improved immune function. The presence of Bacteroidetes may contribute to a favorable CD4/CD8 balance by promoting a healthier gut microbiome and supporting immune regulation, thus aiding in the control of viral reactivation [165,166].

Since *Romboutsia* and *Roseburia* are known SCFA producers, their metabolites could exert immunomodulatory effects that influence the immune response to BKPyV and impact viral replication and reactivation. Notably, *Roseburia* and *Blautia*—members of the phylum Firmicutes, class Clostridia, family Lachnospiraceae—have been identified as beneficial SCFA producers, supporting intestinal barrier function and serving as key energy sources for epithelial cells [167]. Recent research has revealed that certain gut microbiota, including *Roseburia* and *Blautia*, exhibit a negative association with CD4^+^ T cell counts and a positive association with CD8^+^ CD57^+^ T cell levels. These findings suggest that these microbial populations may modulate immune responses against viral infections and reactivation, potentially influencing treatment outcomes [168].

Immunosuppression, whether due to medications following organ transplantation or to underlying conditions such as HIV/AIDS, significantly increases the risk of viral reactivation, including BKPyV [169,170]. In immunocompromised individuals, the capacity to mount a robust BKPyV-specific T cell response is often diminished, resulting in viral replication and subsequent nephropathy [154,160]. This failure of cellular immunity arises from mechanisms including impaired CD8^+^ T cell activity and sustained type I interferon (IFN-I) responses, which hinder effective adaptive immune activation and are closely linked to gut microbiota composition.

The gut microbiota therefore plays a crucial role in immune reconstitution following immunosuppression, impacting both innate and adaptive immunity. Dysbiosis can compromise intestinal barrier integrity, allowing microbial antigens to translocate into circulation and triggering systemic immune activation and inflammation. Studies have shown that a reduction in beneficial bacterial species such as *Faecalibacterium* and *Akkermansia*, alongside an increase in pathogenic taxa such as *Clostridium sensu stricto 1*, can impair immune cell recovery—particularly of neutrophils and lymphocytes—following interventions such as bone marrow transplantation [171]. This microbial imbalance fosters chronic inflammation by promoting pro-inflammatory cytokines (e.g., IL-1, IL-6, TNF-α) while reducing anti-inflammatory mediators like IL-10 [93,172]. Such disruptions may trigger autoimmune processes and further exacerbate immune dysregulation, creating a vicious cycle that promotes viral reactivation [93,172].

Additionally, antibiotic treatment—while essential for combating bacterial infections and preventing viral reactivation—can significantly disrupt gut microbiota by decreasing the diversity and abundance of commensal bacteria [173]. Antibiotics often reduce beneficial taxa such as *Lactobacillus* and SCFA-producing species, while favoring the proliferation of pathogenic or antibiotic-resistant strains like *Enterococcus* and *Clostridioides difficile*. These alterations impair key metabolic functions, immune regulation, and gut barrier integrity, thereby increasing susceptibility to infections and viral reactivation, including polyomavirus infections. However, further studies are warranted to fully elucidate these mechanisms [174].

## 5. Microbial Biomarkers for the Prediction of Risk/Susceptibility to Polyomavirus Infections

As previously discussed, the gut microbiota plays a critical role in the regulation of host immunity. Emerging evidence highlights the profound impact of microbial metabolites on immune modulation [90,175]. These metabolites influence several key processes, including the promotion of autophagosome formation, enhancement of neutrophil recruitment and cytolytic activity, suppression of proinflammatory cytokine production by macrophages via histone deacetylase inhibition, and epigenetic reprogramming of Tregs to expand their population [176].

Healthy commensal microbiota, through both direct antimicrobial effects and modulation of host immunity, serves as a key barrier against a wide range of pathogenic infections, both within and beyond the gastrointestinal tract [134,177,178]. Nonetheless, the commensal microbiota can also paradoxically facilitate viral infections through several mechanisms [103]. One such mechanism involves promoting viral gene recombination, thereby increasing viral infectivity and adaptability [179]. Additionally, the microbiota can modulate host immune responses, creating immunoregulatory environments that favor viral persistence. This is achieved, for example, through the induction of IL-10 production by regulatory T cells and the suppression of proinflammatory cytokines such as IFN-γ and tumor necrosis factor-alpha (TNF-α) [180,181,182].

Conversely, certain commensal populations enhance host antiviral defenses by stimulating the production of molecules such as type I interferons [183]. An expanding body of literature has documented how various viral infections—notably hepatitis B virus (HBV) [184], hepatitis C virus (HCV) [185], and severe acute respiratory syndrome coronavirus-2 (SARS-CoV-2) [186,187]—can both alter and be influenced by the intestinal microbiota. Given these parallels, similar microbiota-mediated mechanisms are likely to operate in the context of polyomavirus infections.

Consistent with this notion, a study conducted by Zhang et al. reported a significant increase in the F/B ratio in renal transplant patients infected with BKPyV [90]. This dysbiotic signature mirrors patterns observed in other infections, including HBV [188], human immunodeficiency virus (HIV) infection [189], and Clostridium difficile infections [190]. In this study, the gut microbiota of 25 renal transplant recipients with BKPyV infection was compared to that of 23 matched controls using 16S ribosomal RNA gene amplicon sequencing. The analysis revealed significant dysbiosis within the infected group, prominently characterized by an elevated F/B ratio [90]. Moreover, nine bacterial taxa were identified as potential biomarkers for BKPyV infection, as summarized in Table 2.

Notably, the order *Clostridiales*, a major component of the class *Clostridia*, includes several butyrate-producing taxa such as *Peptostreptococcaceae*, *Veillonellaceae*, and the genus *Romboutsia*. These microbial groups may influence BKPyV infection through their immunomodulatory properties, as the butyrate they produce functions as an inhibitor of HDACs, thereby reducing the production of pro-inflammatory mediators such as interleukin-6 (IL-6), IL-12, and nitric oxide [191]. In parallel, butyrate promotes the secretion of the anti-inflammatory cytokine IL-10 via activation of G-protein-coupled receptors (GPR109a and GPR43), while also suppressing Th17-mediated inflammatory responses [192]. Conversely, the genus *Enterococcus*—which includes bacterial species known to induce or exacerbate inflammatory responses—was found to be significantly reduced in the fecal samples of patients with BKPyV infection. A deficiency in *Enterococcus* abundance may thus serve as a potential predictive biomarker for polyomavirus infection [90,191,193,194].

Building on these observations, the authors developed a predictive algorithm using a random forest model to evaluate the infectious state of BKPyV in renal transplant patients based on their intestinal microbiota profiles [90]. Random forest models are particularly advantageous for predicting algorithm development due to their high accuracy, robustness, and capacity to handle missing or incomplete data. In this study, the random forest classifier, constructed from the identified bacterial genera, achieved an accuracy of 80.71% in predicting BKPyV infection status. These results underscore the potential utility of specific gut microbiota signatures as biomarkers for early detection and longitudinal monitoring of BKPyV infections in transplant recipients.

Overall, these findings highlight the intricate interplay between gut microbiota composition and immune modulation in the setting of renal transplantation, suggesting that microbial profiling could serve as a valuable tool for the clinical management of post-transplant infections [90,195].

## 6. Conclusions

Polyomavirus infections, particularly in immunocompromised individuals, pose a complex clinical challenge influenced not only by immune status, but also by the gut microbiota. Increasing evidence indicates that microbiota-derived metabolites, especially SCFAs, modulate key immune pathways that can influence viral latency, replication, and immune escape [196]. Conversely, polyomavirus infection itself may induce dysbiosis, thereby further destabilizing immune equilibrium [90]. Identifying microbiota-based biomarkers and restoring microbial balance represent promising strategies to enhance the management of polyomavirus-associated diseases. These findings support the integration of microbiota-targeted interventions into current therapeutic frameworks, especially in transplant medicine and other immunosuppressive settings.

## 7. Future Perspective: Therapeutic Potentials of Microbiota Manipulation in Polyomavirus-Associated Diseases

At present, no antiviral medications have demonstrated consistent clinical efficacy against polyomaviruses. Consequently, the management of these infections relies largely on immunotherapeutic approaches and supportive care. Given the intricate relationship between the microbiota and the immune system—both of which significantly influence polyomavirus pathogenesis—the therapeutic manipulation of the microbiota is emerging as a promising area of research. These strategies aim to modulate immune responses through microbiota-centered mechanisms.

Fecal microbiota transplantation (FMT), also known as fecal bacteriotherapy, has been proposed to restore healthy gut microbiota in patients with dysbiosis, thereby potentially improving immune responses to viral infections. FMT involves the administration of fecal matter form healthy donors to replenish the recipient’s microbial diversity [197]. By restoring microbial homeostasis, this intervention may improve immune modulation and enhance the efficacy of antiviral therapies, including those targeting polyomavirus infections.

In May 2013, the U.S. Food and Drug Administration (FDA) classified FMT as an investigational new drug (IND) due to the absence of large-scale phase III clinical trials confirming its safety and efficacy. However, the FDA subsequently exercised enforcement discretion, allowing the use of FMT without IND qualifications for specific indications. Despite its promise, FMT carries inherent risks, including the potential transmission of pathogens from donor to recipient [198]. This concern is particularly relevant to polyomaviruses, which are widely prevalent in the general population and may be inadvertently transferred via donor stool.

Indeed, BK virus has been detected in the stool of healthy individuals, particularly children, and JC virus has been found in approximately 9.1% of adult stool samples [199,200]. These findings highlight the need for stringent donor screening protocols and long-term safety studies, particularly in immunocompromised populations such as renal transplant recipients. Controlled trials are necessary to determine whether FMT or similar microbiota-based therapies can inadvertently contribute to polyomavirus-associated complications.

Despite the known correlation between polyomaviruses and the intestinal microbiota, there is currently no standardized, widely accepted diagnostic protocol for accurately tracking and monitoring the intestinal microbiome in individuals affected by polyomavirus-related diseases. The biological complexity of the microbiome, its interindividual heterogeneity, and the absence of standardized analytical methods remain significant barriers to the clinical integration of microbiome studies—particularly those concerning intestinal viruses such as polyomaviruses. Changes in gut microbiota composition—especially in bacterial taxa or their metabolic activity—can signal systemic immune shifts that may facilitate viral reactivation [201]. In the case of MCPyV, such microbial biomarkers could serve as early indicators of increased viral activity, especially in immunocompromised tissues like the anophthalmic conjunctiva. Incorporating microbiome profiling into clinical practice offers a non-invasive, longitudinal tool for tracking microbial fluctuations through stool analysis, which could be correlated with MCPyV levels at peripheral sites. This integrated approach may enhance early detection of viral reactivation and inform clinical decision making.

Beyond diagnostics, integrating microbiome data with virological parameters could improve risk stratification for vulnerable populations—such as transplant recipients or individuals with HIV—and support more personalized monitoring strategies. Furthermore, identifying microbial signatures associated with immune resilience could pave the way for microbiome-based therapies. Interventions such as probiotics, prebiotics, or synbiotics may help modulate the microbiota to suppress viral replication [202]. These approaches align with the objectives of precision microbiome medicine, which seeks to leverage host–microbiota interactions for managing persistent viral infections.

Given these limitations, alternative microbiota modulation strategies are receiving increased attention. Probiotics—live microorganisms that confer health benefits—and prebiotics—dietary substrates that promote the growth of beneficial microbes—are being investigated as safer and more targeted options. Both have demonstrated potential to restore gut microbial balance and support immune function. Probiotics, in particular, can enhance the production of SCFAs, anti-inflammatory metabolites that may protect against viral-induced immune overactivation and tissue damage [203]. Dietary interventions, such as increasing the intake of fiber-rich foods, represent another avenue for microbiota modulation. Dietary fibers—including polysaccharides (e.g., cellulose, pectins), oligosaccharides, and lignins—are fermented by the gut microbiota in the colon, producing SCFAs and other metabolites that support immune regulation [204]. Higher fiber consumption has been associated with increased SCFA production, improved immune function, and potentially more favorable outcomes in the case of viral infections, including those involving polyomaviruses [205].

Overall, diet plays a pivotal role in shaping the gut microbiota and may be strategically leveraged in the prevention and treatment of polyomavirus infections [206]. Evaluating how dietary components influence microbial composition, metabolite production, and host immune responses remains a promising and active area of research.

In addition, emerging synthetic biology approaches are being explored to design targeted microbial consortia or engineer metabolites that can inhibit viral replication or enhance antiviral immunity [207]. These next-generation strategies aim to deliver highly specific, controllable interventions for microbiota-based therapy.

Future research should focus on elucidating how specific microbiota alterations influence immune responses and viral dynamics in polyomavirus-associated diseases. A better understanding of these interactions will be critical for developing microbiota-targeted therapeutics. Moreover, integrating gut microbiome profiling into the diagnostic workflow for polyomavirus-related conditions could improve infection tracking and help tailor treatment strategies.

In summary, microbiota manipulation—whether via FMT, probiotics, dietary strategies, or synthetic microbiota engineering—holds substantial promise as a novel therapeutic approach to enhance antiviral defenses and improve clinical outcomes in polyomavirus-related disorders. Continued investigation will be essential to translate these findings into safe and effective clinical interventions.

## Figures and Tables

**Table 1:** Summary of major human polyomaviruses and their tissue tropism.

Virus	Human Tissue Tropism	Reference
BK Polyomavirus (BKPyV)	Salivary gland cells, peripheral blood leukocytes, pancreatic cells, vascular endothelial cells, upper respiratory tract, and tonsils.	[7]
JC Polyomavirus (JCPyV)	Tonsillar stroma, B cells, kidney cells, oligodendrocytes, astrocytes, glial precursors, and CD34+ hematopoietic precursors.	[8]
KI Polyomavirus (KIPyV)	Respiratory tract, stools, tonsils, and blood cells.	[4]
WU Polyomavirus (WUPyV)	Respiratory epithelial cells and respiratory tract secretions.	[9]
Merkel Cell Polyomavirus (MCPyV)	Merkel cells; keratinocytes and dermal fibroblasts.	[10]
Trichodysplasia spinulosa virus (TSPyV)	Inner root sheath cells of hair follicles, endothelial cells, respiratory tract, tonsils, and blood cells.	[11]
MW Polyomavirus/Human Polyomavirus 10 (HPyV10)	Gastrointestinal–gastroenteric.	[12]
HPyV6 and HPyV7	Skin biopsies and thymic hyperplasia samples.	[13]
HPyV9	Serum; kidney cells and skin cells.	[14]
HPyV12	Liver cells; rectum and colon cells.	[15]
Saint Louis Polyomavirus (STLPyV)	Gastrointestinal.	[16]
Simian virus 40 (SV40)	Astrocytes, lymphocytes, and mesothelial cells.	[17]
New Jersey Polyomavirus (NJPyV)	FFPE muscle tissue.	[18]

**Table 2:** Potential bacterial biomarkers of polyomavirus infection.

#	Taxon	Taxonomic Level	Observed Variation in BKPYV-Infected Patients	Notes
**1**	*Romboutsia*	Genus	Significantly increased abundance	Most prominent change
**2**	*Actinomyces*	Genus	—	Included in the predictive model
**3**	*Clostridia*	Class	Increased abundance	
**4**	*Clostridiales*	Order	Elevated levels	
**5**	*Peptostreptococcaceae*	Family	Higher abundance	
**6**	*Veillonellaceae*	Family	More prevalent	Particularly enriched in the infected cohort
**7**	*Enterococcaceae*	Family	Decreased abundance	Reduced in BKPYV-infected individuals
**8**	*Enterococcus*	Genus	Lower levels compared to controls	
**9**	Uncultured bacterium (genus *Romboutsia*)	Genus (uncultured)	Increased abundance	Also highlighted as a key indicator

## Data Availability

Not applicable.

## Abbreviations

The following abbreviations are used in this manuscript:

BKPyV	BK polyomavirus
BKPyVAN	BKPyV-associated nephropathy
cGAS	Cyclic GMP-AMP synthase
CNS	Central nervous system
CNS1	Conserved non-coding sequence 1
CRC	Colorectal cancer
DCs	Dendritic cells
dsDNA	Double-stranded DNA
F/B	Firmicutes/Bacteroidetes
FDA	Food and Drug Administration
FMT	Fecal microbiota transplantation
GABA	Gamma-aminobutyric acid
GALT	Gut-associated lymphoid tissue
GBA	Gut–brain axis
GLP-1	Glucagon-like peptide-1
GVHD	Graft-versus-host disease
HBV	Hepatitis B virus
HCV	Hepatitis C virus
HDAC	Histone deacetylase
HIV	Human immunodeficiency virus
HLA	Human leukocyte antigen
HPyV10	Human polyomavirus 10
HPyV12	Human polyomavirus 12
HPyV6	Human polyomavirus 6
HPyV7	Human polyomavirus 7
HPyV9	Human polyomavirus 9
IBD	Inflammatory bowel disease
IFN-I	Type I interferon
IFN-β	Interferon-β
IFN-γ	Interferon-γ
IL-10	Interleukin-10
IL-12	Interleukin-12
IL-18	Interleukin-18
IL-2	Interleukin-2
IL-6	Interleukin-6
IND	Investigational new drug
IRF3	Interferon regulatory factor 3
JCPyV	JC polyomavirus
KIPyV	KI polyomavirus
LI	Large intestine
LPS	Lipopolysaccharides
LT	Large T antigen
MCPyV	Merkell cell polyomavirus
MNPs	Mononuclear phagocytes
MPyV	Murine polyomavirus
NCCR	Noncoding control region
NF-κB	Nuclear factor-κb
NJPyV	New Jersey polyomavirus
NK	Natural killer
PRRs	Pattern recognition receptors
PVR	Polyomavirus receptor
RIG-I	Retinoic acid-inducible gene I
SARS-CoV-2	Severe acute respiratory syndrome coronavirus-2
SCFAs	Short-chain fatty acids
SI	Small intestine
sIgA	secretory immunoglobulin A
sT	small T antigen
STLPyV	Saint Louis polyomavirus
SV40	Simian virus 40
Th	T helper cells
TLR9	Toll-like receptor 9
TLRs	Toll-like receptors
TMAO	Trimethylamine N-oxide
TNF-α	Tumor necrosis factor-alpha
Tregs	Regulatory T cells
TSPyV	Trichodysplasia spinulosa virus
WUPyV	WU polyomavirus
